# A two-phase approach to re-calibrating expensive computer simulation for sex-specific colorectal neoplasia development modeling

**DOI:** 10.1186/s12911-022-01991-7

**Published:** 2022-09-18

**Authors:** Carolina Vivas-Valencia, You Zhou, Aditya Sai, Thomas F. Imperiale, Nan Kong

**Affiliations:** 1 Weldon School of Biomedical Engineering, Martin C. Jischke Hall of Biomedical Engineering, Purdue University, 206 S. Martin Jischke Drive, West Lafayette, IN 47907-2032 USA; 2 Carbon Health, San Francisco, CA USA; 3 Indiana University School of Medicine, Indiana University, Indianapolis, IN USA; 4 Richard A. Roudebush VA Medical Center, Indianapolis, IN USA; 5 Regenstrief Institute, Indianapolis, IN USA

**Keywords:** Computationally expensive simulator, Colorectal neoplasia, State-transition model, Model recalibration

## Abstract

**Background:**

Medical evidence from more recent observational studies may significantly alter our understanding of disease incidence and progression, and would require recalibration of existing computational and predictive disease models. However, it is often challenging to perform recalibration when there are a large number of model parameters to be estimated. Moreover, comparing the fitting performances of candidate parameter designs can be difficult due to significant variation in simulated outcomes under limited computational budget and long runtime, even for one simulation replication.

**Methods:**

We developed a two-phase recalibration procedure. As a proof-of-the-concept study, we verified the procedure in the context of sex-specific colorectal neoplasia development. We considered two individual-based state-transition stochastic simulation models, estimating model parameters that govern colorectal adenoma occurrence and its growth through three preclinical states: non-advanced precancerous polyp, advanced precancerous polyp, and cancerous polyp. For the calibration, we used a weighted-sum-squared error between three prevalence values reported in the literature and the corresponding simulation outcomes. In phase 1 of the calibration procedure, we first extracted the baseline parameter design from relevant studies on the same model. We then performed sampling-based searches within a proper range around the baseline design to identify the initial set of good candidate designs. In phase 2, we performed local search (e.g., the Nelder-Mead algorithm), starting from the candidate designs identified at the end of phase 1. Further, we investigated the efficiency of exploring dimensions of the parameter space sequentially based on our prior knowledge of the system dynamics.

**Results:**

The efficiency of our two-phase re-calibration procedure was first investigated with CMOST, a relatively inexpensive computational model. It was then further verified with the V/NCS model, which is much more expensive. Overall, our two-phase procedure showed a better goodness-of-fit than the straightforward employment of the Nelder-Mead algorithm, when only a limited number of simulation replications were allowed. In addition, in phase 2, performing local search along parameter space dimensions sequentially was more efficient than performing the search over all dimensions concurrently.

**Conclusion:**

The proposed two-phase re-calibration procedure is efficient at estimating parameters of computationally expensive stochastic dynamic disease models.

**Supplementary Information:**

The online version contains supplementary material available at 10.1186/s12911-022-01991-7.

## Background

Colorectal cancer (CRC) is the second most common cancer in the United States for men and women combined [1]. In 2017, there were roughly 135,000 new CRC cases, with 45% of men and 39% of women younger than 65 at the age of diagnosis [2]. Colorectal neoplasia development can take many years, remaining asymptomatic for much or all of this time. The development starts with small pre-cancerous polyps growing in the internal lining of the colon and rectum. These polyps may gradually increase in size or develop advanced histological features. Finally, advanced, precancerous polyps may evolve into invasive adenocarcinoma, eventually spreading locally or systemically through lymph and blood vessels. The five-year survival rate of CRC is 90% when the cancer is confined to the colon and rectum, whereas the five-year survival rate declines to 12% when it has spread to distant locations [3].

For CRC prevention, along with improving the accuracy and convenience of screening tests, there is a need to improve the prediction of tumor incidence and symptom onset via computational and predictive modeling of colorectal neoplasia development. Once this need is addressed, diagnostic screening and surveillance can be better targeted on those at high risk of rapid disease progression. In recent clinical practice, patients are often further classified by detection of advanced precancerous polyps, which include adenomas and sessile serrated polyps ≥ 10 mm, and adenomas with villous histology or high-grade dysplasia [4, 5]. Individuals with advanced precancerous lesions are more likely to develop other advanced lesions and asymptomatic CRC [6]. With improved prediction of precancerous lesion advancement, population surveillance can become more effective and cost-effective.

Additional evidence from more recent clinical studies may emerge, which requires updates on our understanding of colorectal neoplasia development. Moreover, many of these studies are conducted by exploring risk factors, e.g., comparing men and women, on the development. As a result, we often need to retrain existing computational models (i.e., updating the estimates of model parameters) to quantify the differences based on new data from the same population and/or data from a new population with distinct features from previously studied ones. This can help provide predictive intelligence on timely adjustment of CRC screening and surveillance strategies in terms of cost-effectiveness [7–13]. However, the computational models tend to become much more expensive with the incorporation of additional risk factors. Therefore, there is a need to develop an efficient algorithmic procedure for re-calibrating expensive computational models.

This paper used the predictive modeling of sex-specific colorectal neoplasia development in a proof-of-the-concept study. We adapted two independently developed and well-established CRC disease models, both of which are individual-based state-transition models [15, 22]. Due to CRC-related behavior changes and clinical interventions (e.g., polypectomy) available, real-world patient medical records cannot provide sufficient age- and sex-specific incidence information about colorectal neoplasia development under natural circumstances. In response, we resorted to model calibration against sex-specific prevalence data on key CRC preclinical stages. Note that there are multi-year population surveillance studies that collect colonoscopy images and derive sex-specific prevalence on the preclinical natural history, e.g., Brenner et al. [14].

Only a handful of papers in the CRC computational modeling literature have reported their model calibration work in detail. Roberts et al. [15] developed the V/NCS model, a discrete-event simulation model used in the current study, on a self-created object-oriented simulation platform, with a focus on the modeling of CRC events and the event relationships. The authors reported in their prior manuscripts (e.g., [16–18]) a series of model calibration activities through heuristics against epidemiological adenoma prevalence and CRC incidence data. Erenay et al. [19] developed an individual-based event-driven state transition simulation that mimics the natural history of metachronous colorectal cancer (MCRC) for a 5-year period following the treatment of primary CRC. The model comprises five states, namely polyp-free, polyp, MCRC, metastatic-MCRC, and MCRC-related death. The authors estimated six unknown parameters of the natural history of MCRC through calibrating the simulation mentioned above against two calibration targets, namely 5-year MCRC incidence and mortality rate, with the principle of least sum-of-squared error. For the calibration, the authors simply ran the simulation model exhaustively with every possible combination of the unknown parameters and selected those with simulated outputs matching the benchmark statistics of a well-defined patient cohort, derived from the SEER database. Rose et al. [20] proposed an individually-based state transition model consisting of two interacting submodels, namely a continuous-time disease-progression submodel and a discrete-time Markov submodel for surveillance and retreatment. The key components for modeling the disease progression are recurring transitions to unresectability and symptom onset, either of which is determined by a transition timing and modeled with an exponential distribution. The author estimated seven unknown parameters of disease progression through calibrating the simulation mentioned above against seven observable outcomes, reported in Pietra et al. [21]. The authors developed a calibration procedure that consists of several rounds of calibration with increasingly narrowed candidate parameter sets and against a series of calibration targets. Prakash et al. [22] developed the CMOST model, an open-source framework for the microsimulation of CRC screening strategies also used in our study, facilitating automated parameter calibration against epidemiological adenoma prevalence and CRC incidence data. The authors used a heuristic greedy algorithm followed by Nelder-Mead optimization [23] to minimize the squared error between the benchmark values and the corresponding model predictions.

Sai et al. [24] investigated the efficiency of a Gaussian Processes-based surrogate modeling approach to approximate the CMOST model to alleviate the computational burden in calibrating the CMOST model. Compared to above papers in the literature, we studied a different version of the calibration problem, for which we have the option of using a baseline parameter design from the literature and/or previous studies to start the model parameter adjustments. In addition, we conducted comparative studies on the effect of global search as the predecessor of the Nelder-Mead optimization and compared different settings of Nelder-Mead to further improve the calibration efficiency.

It is evident that sex plays an essential role in CRC incidence and progression, in addition to a wide range of risk factors, including family history [25] and lifestyle-related ones such as smoking [26], red-meat diet [27], among other factors [28–30]. More men than women are diagnosed with CRC. While men and women have similar genetic predispositions, there are substantial differences in CRC incidence between the two sexes [31, 32]. In addition, several studies suggest that females diagnosed with CRC have significantly longer survival than males [33, 34]. Further, men have a higher prevalence of adenomas than women. For example, Ferlitsch et al. [35] reported that adenomas prevalence was higher among men than women by an absolute difference of 10%, studying more than 44 thousand participants in a national screening colonoscopy program in Austria. In a study of more than 50,000 Polish participants, Regula et al. [36] reported that advanced precancerous polyp was found with a significantly higher percentage in men than women. Brenner et al. [14] reported that adenoma prevalence (both advanced and non-advanced) was substantially higher in men than in women for different age groups, from an observational study of more than 3.6 million German participants. Different from the above observational studies, we applied computational and predictive modeling to differentiate colorectal neoplasia development between the two sexes and over age groups.

From the above literature review, we concluded that existing studies have not addressed several challenges in modeling and model calibration of CRC natural history and beyond. The main contribution of the current study is the development of an efficient re-calibration procedure for expensive stochastic simulations of disease natural history. We believe our method works well on all kinds of individual-based state-transition disease models with a high-dimensional model parameter space, unbounded value range on each parameter, some prior knowledge on the association among different parameters, and expensive computational simulation run. Further, through our proof-of-the-concept study, we quantified the age-dependent sex differences in colorectal neoplasia development.

## Methods

We proposed a two-phase procedure to re-calibrate computationally expensive disease simulations whose features have been described previously. In phase 1, we performed global sampling to identify reasonably good candidate parameter designs. In phase 2, we performed local search to further improve model fitting. To efficiently adapt the local search idea, we compared two variants of the Nelder-Mead algorithm implementation (i.e., exploring subsets of parameter space dimensions sequentially vs. exploring the entire parameter space concurrently). We termed the two variants *axial-based search* and *global search*, respectively.

To investigate the procedure efficiency, we adapted two individual-based state-transition CRC natural history models as test cases. We set a weighted-sum-squared-error on the prevalence of three preclinical disease states as the loss function to minimize and used benchmark statistics extracted from a German cohort study by Brenner et al. [14]. Brenner and colleagues analyzed national screening colonoscopy registry data from nearly 3.6 million German participants, qualifying the prevalence of each lesion in 5-year age groups. As a byproduct, we quantified the sex-specific colorectal neoplasia development in different age groups.

## Overview of the disease models

CRC begins with colorectal precancerous polyps, either adenomas or sessile serrated lesions. For ease of terminology, we shall refer to any precancerous polyp as an adenoma. After the occurrence of an adenoma, it gradually transitions to next stages, depending on the pathway to cancer it follows. Our study utilized the CMOST and V/NCS models, two well-known individual-based state-transition models of CRC. Both models share commonalties at the conceptual level in terms of the neoplasia development process. Both models, in principle, can simulate adenoma occurrence and then sequentially going through non-advanced stage, advanced stage, and eventually becoming invasive cancer prior to clinical stages, without reversing events. Nevertheless, it is worth pointing out that both models are expansive at investigating the efficacy of screening methods and strategies, which requires to delay or even reverse of the adenoma progression due to, for example, polypectomy.

The two models share key assumptions regarding adenoma occurrence and growth. Most relevant to our work is that occurrence and growth of each adenoma are independent of other adenomas. Nevertheless, the two models differ in the quantification of the state transitions. The CMOST model incorporates age-dependent adenoma occurrence and adenoma-specific growth rates to specify the state transition probabilities for each individual adenoma along the adenoma-carcinoma sequence. All the transitions are modeled instantaneously in time increments of 3 months. While the V/NCS model does not incorporate age-dependency in modeling the adenoma occurrence and growth, it can differentiate individuals’ neoplasia development by several known risk factors, including age, sex, race, and family history. In addition, V/NCS models the growth of each adenoma with its dwell duration (i.e., length of time spent) at a state. Thus, the transition events are scheduled in reference to a simulation clock and thus they take place according to the specified timing in the simulation. In the V/NCS model, we also associated the simulated cohort with year-specific mortality risks. For more information, please see Additional file 1: Appendix A.

In summary, there are 19 unknown parameters in the CMOST model and 8 unknown parameters in the V/NCS model. For lists of the parameters, please see Additional file 2: Appendix B.

## Simulator adaptation for the calibration

**Fig. 1:**
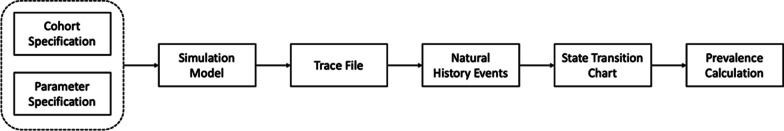
Illustration of simulator adaptation

With either model platform, one can input a set of characteristics necessary to define a cohort of some arbitrary size, e.g., one that matches the U.S. population. The simulation can then trace colorectal neoplasia development of each individual in the cohort. Note that as an addition in the V/NCS model, the start and end years of the simulation can be specified to match with the reported year-specific US census. One beneficial feature of both model platforms is that they can generate a trace statement that summarizes a sequence of periodic transitions directly (for the CMOST model) and time-stamped events (for the V/NCS model). One can use the trace statement to calculate the state-specific prevalence values (population distribution among the three disease stages – NON, ADV, and CRC) and thus capture the neoplasia development. More specifically, we developed a procedure to extract a state transition chart for each simulated individual. By following each individual through the simulation duration, one can characterize her disease stage at any specific point. For each of the five age groups (54–59, 60–64, 65–69, 70–74 and 75–79), we counted its population at the end of the simulation horizon and calculated the portion of the corresponding population subgroup in each of the three states as the corresponding prevalence value. The simulation adaptation is summarized as Fig. 1.

In the CMOST model, state NOV represents the subpopulation at early adenoma stages I-IV; state ADV represents the subpopulation at advanced adenoma stages V, VII; and state CRC represents the subpopulation with preclinical and clinical cancer. In the V/NCS model, state NOV includes individuals who have had at least one progressive or non-progressive non-advanced adenoma, or at least one adenoma that immediately progresses to cancer; state ADV includes those who have had at least one advanced adenoma but none has become cancerous; and state CRC includes those who have had cancerous adenomas or have developed CRC.

## A two-phase calibration procedure

We proposed a two-phase approach to the model calibration. In phase 1 (a preliminary phase), we performed global searches in an ad-hoc manner. When the computational experiment is expensive, we elected to cluster the parameters based on their physical meanings and performed the searches progressively against aggregate calibration targets over age groups. The purpose was to identify a promising parameter design as the starting point for phase 2. In phase 2, we viewed the model calibration task as a nonlinear optimization problem. We performed the Nelder-Mead algorithm (simplex search algorithm), one of the best-known algorithms for multidimensional unconstrained optimization without derivatives. Given the high-dimensionality of the “black-box” optimization problem, we explored two variants of the search procedure, namely: (1) one-shot globally over the entire model parameter space, and (2) sequentially based on interconnections in subsets of model parameters. We provide more details in the following.

### Phase 1 (Preliminary phase): identify promising initial search points for Phase 2

For the CMOST model, since the computational burden is much less, we can directly identify the promising values for all 19 input model parameters. For each parameter with baseline value $v_{i}$, we extended it to a range with ± 20%, i.e., $\left[ {0.8v_{i} ,1.2v_{i} } \right]$. We applied Latin Hypercube Sampling to select 100 designs randomly from these ranges. Then we ran CMOST with the 100 designs and returned the first design satisfying the criterion that for the three system responses, namely the relative errors are all within 10% to the calibration targets. This design will be the initial point for Phase 2.

For the V/NCS model, since the computational burden is much more, we took caution and consulted the domain expert to finalize each search range, which is also centered around the baseline value. Through our preliminary simulation analysis, we observed that in each pair of *δ* and *γ*, the prevalence values are a lot more sensitive to changes in *δ* than in *γ*. Thus, we set a larger range for each *δ* than the paired *γ*. We divided the search subspace of (*δ*_0_, *γ*_0_) with a five-by-five grid and divided each of *δ*_1_, *γ*_1_, *δ*_2_, *γ*_2_, *δ*_3_, *γ*_3_ with ten even intervals. We followed the adenoma-carcinoma sequence to calibrate the model parameters progressively, i.e., first adenoma progression propensity, then transition from NON to ADV, and finally transition from ADV to CRC. In the first step, we performed a grid search on (*δ*_0_, *γ*_0_) and fixed the other parameter values as one should first carefully emulate the adenoma progression risk distribution of the simulated cohort. Our calibration targets are the three prevalence values for each age group. At the end of this step, we identified promising (*δ*_0_, *γ*_0_) values such that the predicted prevalence values are reasonably close to the observations (less than 15% relative error). Next, we fixed (*δ*_0_, *γ*_0_) values to the identified ones and performed orthogonal sampling in the subspace formed by (*δ*_1_, *γ*_1_). The use of a sampling-based search as opposed to a grid search is because multiple promising (*δ*_0_, *γ*_0_) values were identified and thus using all of them for ensuing search would be computationally expensive. Our calibration targets are aggregate prevalence values of NON and ADV over age groups. We then followed the same idea to search in the subspace formed by (*δ*_2_, *γ*_2_) and used the same calibration targets. We perturbed (*δ*_1_, *γ*_1_) first because there were many more transitions from P_NON to ADV than from NP_NON to ADV. At the end of this step, we identified promising (*δ*_1_, *γ*_1_) and (*δ*_2_, *γ*_2_) designs such that both predicted prevalence values (i.e., at states NON and ADV) were further closer to the observations (less than 10% relative error). Finally, we fixed (*δ*_0_, *γ*_0_), (*δ*_1_, *γ*_1_), (*δ*_2_, *γ*_2_) to be the identified values and performed orthogonal sampling in the subspace formed by (*δ*_3_, *γ*_3_). Our calibration targets are aggregate prevalence values of NON, ADV, and CRC over age groups. At the end of this step, we identified promising (*δ*_3_, *γ*_3_) designs such that all three predicted prevalence values fall in a close range of the target values (less than 10% relative error on NON, less than 10% relative error on ADV, and less than 5% relative error on CRC). To facilitate the calibration, we used a built-in interactive visual tool to graph the corresponding Johnson SB distributions. We discarded some of the parameter designs according to the domain expert’s suggestion.

### Phase 2: Local-search based nonlinear optimization

In this phase, we employed the Nelder-Mead algorithm for gradient-free nonlinear optimization to further improve the model fitting. We set the parameter design identified in phase 1 as the starting point for the Nelder-Mead. We used the weighted sum squared of the relative errors on the three aggregate prevalence values as a similarity measure and the objective function of the unconstrained nonlinear optimization problem (i.e., loss function of the calibration variables). Through consulting with our domain expert, we assigned a larger weight to CRC similarities than ADV similarities and NON similarities.

Considering that it takes a long time to evaluate just one parameter design, we designed two search paths that differ by the search space chosen along the solution process. For V/NCS, we considered the entire 8-dimensional search space the solution. We termed this strategy the “full-space local search strategy.” As an alternative option, we considered four subspaces progressively with an order identical to that in phase 1. That is, the responses are more sensitive to (*δ*_0_, *γ*_0_), than (*δ*_1_, *γ*_1_), than (*δ*_2_, *γ*_2_), and finally (*δ*_3_, *γ*_3_). When performing Nelder-Mead in one subspace, others were fixed at the initial values. We termed this strategy the “sequential local search strategy.” For CMOST, we grouped the 19 parameters in 3 groups, namely “adenoma initiation stage”, “early adenoma stage” and “advanced adenoma stage”. We then assigned each parameter to the corresponding group. We followed a similar idea as above to sequentially calibrate parameters from “initiation” to “early” and then to “advanced” stage.

## Results

We set the simulated cohort in both the CMOST and the V/NCS models to be a population of 1000 white males or females with no family history who were born in 1949. In this way, we could utilize additional parameters previously made available in both models. We first examined the efficiency of our two-phase calibration approach to the relatively inexpensive model CMOST, in comparison with a straightforward execution of the full-space Nelder-Mead algorithm. To use the Nelder-Mead algorithm, we simply called the MatLab function (fminsearch) and ran it to termination of the MatLab default setting and under some predefined Search Effort for the maximum number of function evaluations. Comparative results on CMOST showing loss function values with mean (standard deviation) over 20 runs are reported in Table 1.

**Table 1:** Loss function values for calibration strategy comparison with CMOST

Search effort	100	200	500
Straightforward full-space	0.05993 (0.00405)	0.00219 (0.00023)	0.00032 (0.00032)
Two-phase full-space	0.01670 (0.00112)	0.02319 (0.00243)	0.00592 (0.00398)
Two-phase sequential	0.01048 (0.00103)	0.03366 (0.00531)	0.01368 (0.01148)

For two-phase approaches under a search effort of 100 function evaluations, we excluded those evaluations that used up all the search effort just in phase 1

**Fig. 2:**
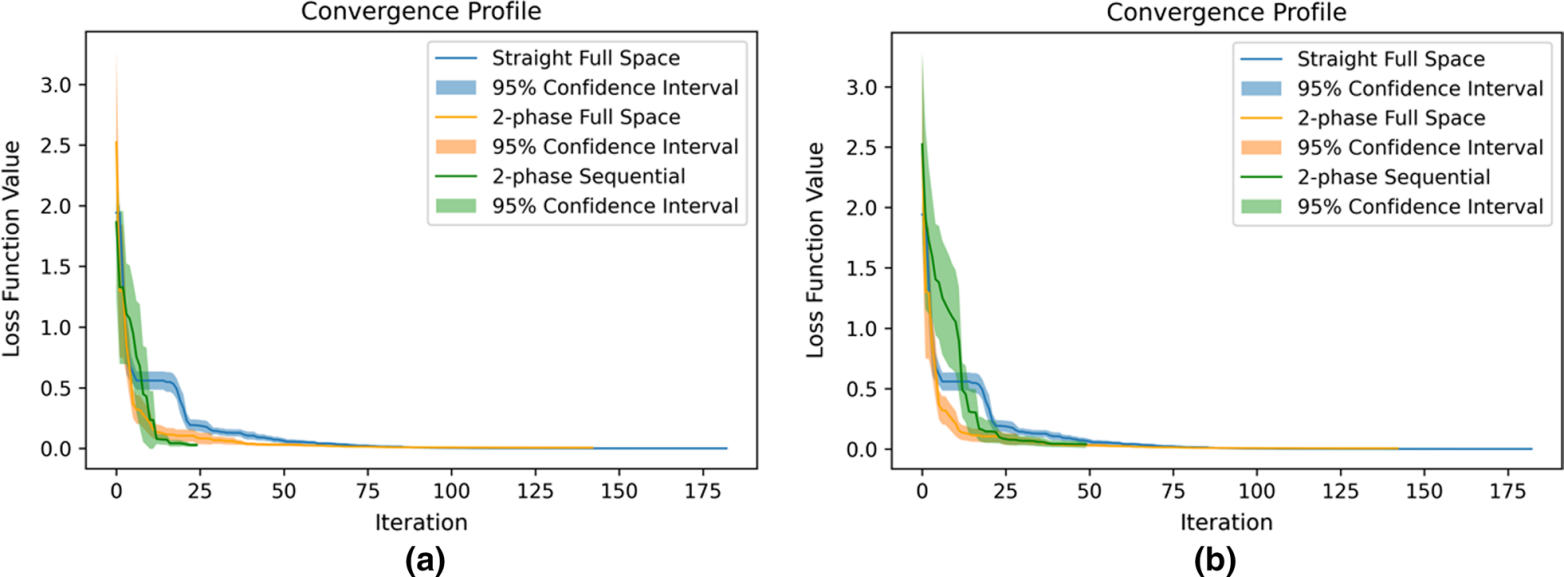
Convergence profiles for the 3 algorithms over 20 runs. **a** 2-Phase sequential method with 100 search effort, **b** 2-phase sequential method with 200 search effort

The comparison between the straightforward approach and the two-phase approach can reveal the effect of the proposed preliminary phase. In the experiment, phase 1 took modest function evaluations to reach a promising initial point, which significantly helped the local search in phase 2 achieve much faster convergence. As shown in Fig. 2, when both applying the two-phase approach, the sequential search strategy with Nelder-Mead in phase 2 could outperform the corresponding full-space search strategy when search effort was limited (i.e., 100 evaluations). Thus, the sequential strategy could further improve the convergence effectively under limited search effort allowed, as long as the parameter subspaces and their relative precedence can be reasonably specified. Moreover, the results over 20 runs showed that all three algorithms behave reasonably robust in calibration of CMOST, a stochastic simulation. Though the two-phase full-space search strategy could yield a better goodness-of-fit when the search effort is relatively significant (i.e. a total of 500 function evaluations allowed), we speculated that its sequential search counterpart would be more efficient when dealing with models that are complex and computationally expensive since the CMOST model takes about 25 s to finish one function evaluation on a personal laptop (or close to 1 h for 100 evaluations), while the V/NCS model can easily take over 30 min to run just one evaluation. We thus performed similar calibration exercises on the V/NCS model.

For the V/NCS model, a more computationally expensive model, we report comparative results in Table 2. Overall, our two-phase approach again showed a better goodness-of-fit than the straightforward Nelder-Mead implementation. For example, for a male cohort, both with the sequential search strategy for Nelder-Mead in phase 2, the two-phase approach yielded a loss function value of 0.0025 whereas the straightforward calibration with Nelder-Mead yielded a loss function value of 0.0251 (ten-fold reduction). The same observation was made when both applied full-space search. For female cohort, the improvement was much more noticeable. Further, when comparing the two local search strategies, the full-space search strategy yielded a lower loss function value than the sequential search strategy (male, 0.0025 vs. 0.0056; female, 0.0005 vs. 0.0008). Additionally, these preliminary results suggested that the two-phase approach was more effective in calibrating the V/NCS model for a female cohort than a male cohort.

**Table 2:** Loss function values for calibration strategy comparison with V/NCS

	Male		Female	
	Sequential	Full-space	Sequential	Full-space
Direct local search	0.0251	0.0465	0.0230	0.2969
Two-phase approach	0.0025	0.0056	0.0005	0.0008

With the above results, we concluded that our two-phase approach with the sequential local search strategy is effective. We next specified the number of simulation replications to be 10 to ensure the statistical confidence on stochastic dominance for each comparison. We collected prevalence statistics for five different age groups over the range of 55–79. Table 3 shows the percentage of people with advanced adenoma for men and women within each of the five age groups. Our results show that the model with calibrated variables underestimated male advanced adenoma prevalence and overestimated female advanced adenoma prevalence for younger age groups, whereas it overestimated male advanced adenoma prevalence and underestimated female advanced adenoma prevalence. On the other hand, the comparative results on the prevalence of adenomas having become cancerous were just the opposite except for the age group 55–59 years. Overall, the results supported our calibration of the V/NCS simulation for sex-specific colorectal neoplasia development modeling.

**Table 3:** Age-specific prevalence for advanced adenoma and cancerous neoplasia

	Percentage (%) people with advanced adenoma				Percentage (%) people with cancerous neoplasia			
	Male	Target	Female	Target	Male	Target	Female	Target
55–59	6.25	6.6	3.85	3.50	0.73	0.60	0.27	0.30
60–64	7.63	8.2	4.98	4.50	0.82	1.00	0.56	0.50
65–69	10.41	9.2	5.38	5.30	0.85	1.30	1.03	0.70
70–74	11.31	9.9	5.76	6.40	2.20	1.90	0.92	1.10
75–79	13.17	10.4	6.71	6.80	2.62	2.50	1.44	1.60

## Discussions and conclusion

In this paper, we introduced an efficient two-phase recalibration approach to estimate parameters in computationally expensive disease natural history models with prior point estimates on the model parameters. As a use case, we took into consideration the adenoma-carcinoma sequence and calibrated large sets of unknown parameters in two CRC natural history models. We quantified the sex- and age-specific adenoma-carcinoma sequence based on observations from a large cohort study. We hope to showcase an essential step in assessing the population-level cost and effectiveness of CRC screening methods and strategies for a population whose prevalence data were recently acquired.

Our study has the following limitations. In response to some of the limitations, we point out future research directions. First, we specified the calibration procedure subjectively at several places, e.g., the stopping criteria for phase 1. Alternatively, we will explore the use of Bayesian calibration. Second, for the weighted sum in the loss function, we resorted to one domain expert, which may not have the buy-in from others. In addition, there was no real understanding on how the specification of weighting coefficients in the loss function affects the calibration results. We will explore the use of multi-objective optimization methods to alleviate the subjectivity concern. Third, using Nelder-Mead in phase 2 could well result in a local minimum. We will investigate the use of genetic algorithms such as NSGA-II, which in fact is intended to deal with optimization problems with multiple objectives. Finally, while we proposed to perform calibration on subsets of parameters sequentially and made a solid effort to identify the relative sensitivity, this procedure in phase 1 for the V/NCS model was by no means of scientific rigor. In addition, the progressive calibration procedure in phase 1 could have brought more valuable insights into efficient calibration for tailored natural history models, if not for limited access to the V/NCS model. We were not able to modify the source code of the simulator.

## Supplementary Information

**Additional file 1**.** Appendix A**. The precancerous pathways (i.e., adenoma occurrence and growth) calibrated in the two models.

**Additional file 2**.** Appendix B**. Summaries of the model parameters to be estimated through calibration.

**Additional file 3**. These two tables present the values of initial guess and the resulting optimized parameters of different approaches for both the CMOST model and V/NCS model.

## Data Availability

We have made the final estimates of model parameters of the two CRC models available. We have also listed the prevalence table from Brenner et al. [14], which is used to generate calibration targets for our work (see Additional file 3).

## Abbreviations

CRC: Colorectal Cancer
CMOST: Colon modeling open source tool
V/NCS: Vanderbilt-NC State simulation model
