# Supplementary material for: A two-phase approach to re-calibrating expensive computer simulation for sex-specific colorectal neoplasia development modeling
Source: BMC Med Inform Decis Mak. 2022 Sep 18;22:244. doi: 10.1186/s12911-022-01991-7 (PMC9482725; doi:10.1186/s12911-022-01991-7)
Supplement: Supplementary file 1 — Additional file 1. Appendix A. The precancerous pathways (i.e., adenoma occurrence and growth) calibrated in the two models. [file 12911_2022_1991_MOESM1_ESM.docx]

**Appendix A. The precancerous pathways (i.e., adenoma occurrence and growth) calibrated in the two models**

**CMOST:**

The CMOST model (Figure A.1) involves three different pathways to preclinical cancer: i) Progression of a stage VI adenoma; ii) Fast progression of a smaller adenoma (stage I-V); and iii) Direct cancer without adenomatous precursors. Altogether 6 distinct adenoma stages are distinguished: Stages I-IV correspond to early adenomas with sizes of 3 mm, 5 mm, 7 mm, and 9 mm, respectively. Stage V corresponds to an adenoma > 1 cm or an adenoma with advanced villous histology, and stage VI corresponds to an adenoma of size > 2 cm.


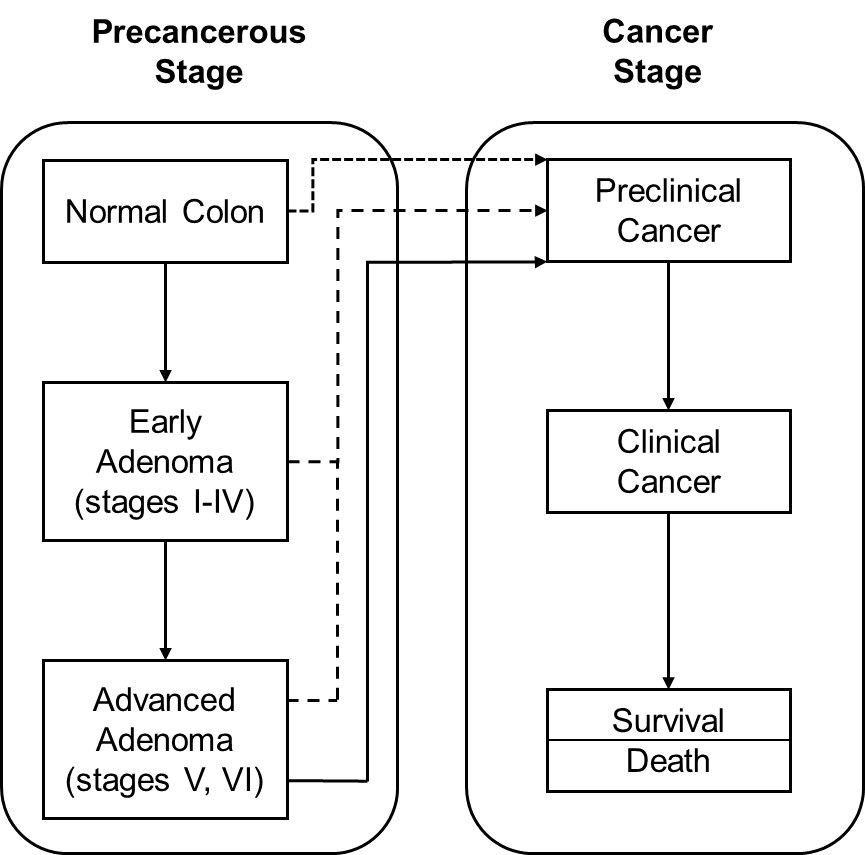


Figure A.1 Transition Diagram in CMOST

The CMOST model can track the history of a general population from birth until death or to a maximum age of 100 years. Adenoma occurrence, growth to advanced adenoma and cancer, cancer staging, as well as CRC screening, and surveillance are all modeled in time increments of 3 months.

For more information about the CMOST model, we refer to Prakash et al. (2017) [1].

**V/NCS:**

The V/NCS model (Figure A.2) involves three pathways to preclinical cancer: non-progressive, slowly progressive, and immediately progressive. The first type is non-progressive. An adenoma of this type has no chance ever to become cancerous, but can grow as a benign adenoma (i.e., advanced adenoma) to match the data on the portion of adenomas that can be detected. The second type is progressive. A non-advanced adenoma of this type can either become an advanced adenoma defined by its histology or become cancerous directly, with the former being more common. The transition of this type is then modeled as a competing process between the above two possibilities. The third type of progression is immediately progressive, which implies that an adenoma with this type immediately progresses to becoming cancerous upon its occurrence. Regardless of the second or third type, as long as an adenoma becomes cancerous, it follows the usual cancer pathway.


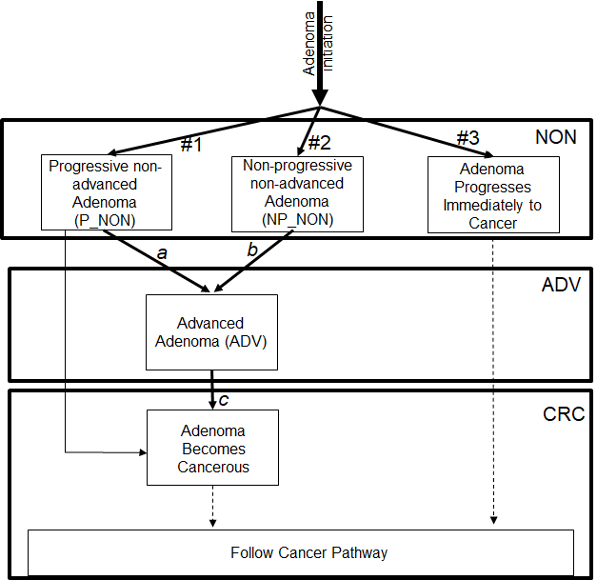


Figure A.2 Transition Diagram in V/NCS

The V/NCS model can track the history of a general population from birth until death or to a maximum age of 120 years. Discrete events like adenoma occurrence and growth, cancer staging, as well as population-level CRC surveillance and individual testing with different methods are all scheduled according to their simulated times.

For more information about the V/NCS model, we refer to Roberts et al. (2007) [2].

**Reference:**

1. Prakash MK, Lang B, Heinrich H, Valli PV, Bauerfeind P, Sonnenberg A, Beerenwinkel N, Misselwitz B. CMOST: an open-source framework for the microsimulation of colorectal cancer screening strategies. BMC Med Inform Decis Mak. 2017;17(1):80.
2. Roberts S, Wang L, Klein R, Ness R, Dittus R. Development of a Simulation Model of Colorectal Cancer. ACM Transactions on Modeling and Computer Simulation. 2007;18(1):4.
