# Supplementary material for: A two-phase approach to re-calibrating expensive computer simulation for sex-specific colorectal neoplasia development modeling
Source: BMC Med Inform Decis Mak. 2022 Sep 18;22:244. doi: 10.1186/s12911-022-01991-7 (PMC9482725; doi:10.1186/s12911-022-01991-7)
Supplement: Supplementary file 2 — Additional file 2. Appendix B. Summaries of the model parameters to be estimated through calibration. [file 12911_2022_1991_MOESM2_ESM.docx]

**Appendix B: Summaries of the model parameters to be estimated through calibration**

**8 parameters in the V/NCS model:**

| **Physical Meaning** | **Unknown Parameter** | **Function Form** |
| --- | --- | --- |
| Individual risk | a JohnsonSB distribution with unknown $\delta_{0}$ and $\gamma_{0}$ | $\gamma_{0}+\delta_{0}log({(X-\theta)}/{(\theta+\sigma-X)}$) |
| Transition time from P_NON to ADV | a JohnsonSB distribution with unknown $\delta_{1}$ and $\gamma_{1}$ | $\gamma_{1}+\delta_{1}log({(X-\theta)}/{(\theta+\sigma-X)}$) |
| Transition time from NP_NON to ADV | a JohnsonSB distribution with unknown $\delta_{2}$ and $\gamma_{2}$ | $\gamma_{2}+\delta_{2}log({(X-\theta)}/{(\theta+\sigma-X)}$) |
| Transition time from ADV to CRC | a JohnsonSB distribution with unknown $\delta_{3}$ and $\gamma_{3}$ | $\gamma_{3}+\delta_{3}log({(X-\theta)}/{(\theta+\sigma-X)}$) |

Note: $\theta$ and $\sigma$ define the range of $X$, where $\theta\leq X\leq\theta+\sigma$. For the individual risk, $\theta=0.0,\sigma=1.0$. For all the transition times, $\theta=0.0,\sigma=60.0$.

**19 parameters in the CMOST model:**

| **Physical Meaning** | **Related Parameter** | **Function Form** |
| --- | --- | --- |
| Age-dependent adenoma initiation rate | a sigmoid function with $\theta_{1},\theta_{2},\theta_{3}$ | $\frac{\theta_{1}}{1+\exp(-(\theta_{2}a-\theta_{3}))}$ |
| Baseline adenoma stage-specific progression rate |  |  |
| Early stages | 4 constants, $\theta_{4}-\theta_{7}$, for Stages I – IV |  |
| Advanced stages | 2 constants, $\theta_{8},\theta_{9}$, for Stages V, VI |  |
| Adenoma-specific progression risk factor |  |  |
| Early stages | an exponential function with $\theta_{10},\theta_{11}$ | $\theta_{10}exp(\theta_{11}p)$ |
| Advanced stages | an exponential function with $\theta_{12},\theta_{13}$ | $\theta_{12}exp(\theta_{13}p)$ |
| Age-dependent progression risk factor |  |  |
| Early stages | a Gaussian function with $\theta_{14},\theta_{15},\theta_{16}$ | $\theta_{14}exp(-{(\theta_{15}a-\theta_{16})}^{2})$ |
| Advanced stages | a Gaussian function with $\theta_{17},\theta_{18},\theta_{19}$ | $\theta_{17}exp(-{(\theta_{18}a-\theta_{19})}^{2})$ |

Note: *p* denotes the risk percentile and *a* denotes the age. Both *p* and *a* are known for the function.
