# Supplementary material for: A two-phase approach to re-calibrating expensive computer simulation for sex-specific colorectal neoplasia development modeling
Source: BMC Med Inform Decis Mak. 2022 Sep 18;22:244. doi: 10.1186/s12911-022-01991-7 (PMC9482725; doi:10.1186/s12911-022-01991-7)
Supplement: Supplementary file 3 — Additional file 3. These two tables present the values of initial guess and the resulting optimized parameters of different approaches for both the CMOST model and V/NCS model. [file 12911_2022_1991_MOESM3_ESM.docx]

These two tables present the values of initial guess and the resulting optimized parameters of different approaches for both the CMOST model and V/NCS model.

**For the CMOST model:**

**For the V/NCS model:**

|  | **Female** | | | | | | **Male** | | | | |
| --- | --- | --- | --- | --- | --- | --- | --- | --- | --- | --- | --- |
|  |  | **Direct Local Search** | | **Two-Phase Approach** | |  | | **Direct Local Search** | | **Two-Phase Approach** | |
|  | **Initial Point** | **Final Point (Sequential)** | **Final Point**  **(Full-Scape)** | **Final Point (Sequential)** | **Final Point**  **(Full-Scape)** | **Initial Point** | | **Final Point (Sequential)** | **Final Point**  **(Full-Scape)** | **Final Point (Sequential)** | **Final Point**  **(Full-Scape)** |
| **Gamma1** | 2.512 | 2.983 | 2.589 | 4.05 | 4.05 | 2.22 | | 2.331 | 2.241 | 2.4615 | 2.4 |
| **Delta1** | 0.622 | 0.5287 | 0.622 | 0.622 | 0.622 | 0.483 | | 0.483 | 0.483 | 0.4031 | 0.4 |
| **Gamma2** | -0.608 | -0.6913 | -0.6265 | -0.3954 | -0.3675 | -1.168 | | -1.226 | -1.150 | -0.53 | -0.5 |
| **Delta2** | 0.521 | 0.521 | 0.5369 | 0.5442 | 0.521 | 0.486 | | 0.5103 | 0.491 | 0.5225 | 0.486 |
| **Gamma3** | 1.108 | 0.6925 | 1.0587 | 0.76 | 0.8 | 1.21 | | 1.702 | 1.221 | 0.9 | 1 |
| **Delta3** | 1.398 | 1.8087 | 1.441 | 1.538 | 1.398 | 1.442 | | 0.8292 | 1.456 | 0.86 | 0.84 |
| **Gamma4** | 0.395 | 0.4148 | 0.3996 | 1.26 | 1.2 | 0.395 | | 0.4246 | 0.3987 | 0.63 | 0.6 |
| **Delta4** | 1.147 | 1.147 | 1.182 | 1.417 | 1.417 | 1.147 | | 1.032 | 1.158 | 0.95 | 1 |


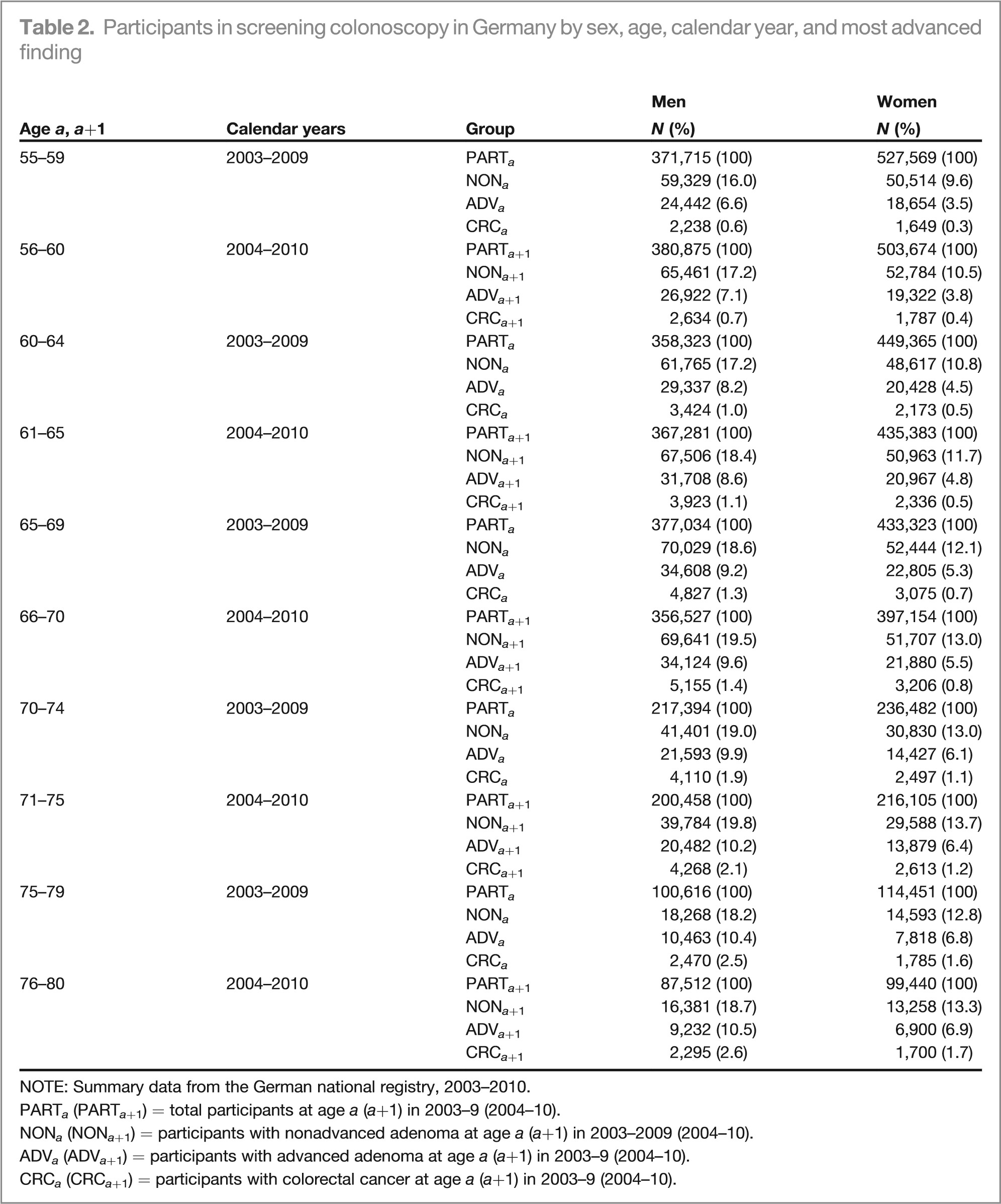
This table from Brenner et al. (2013) [1] provides the total number of screening colonoscopy participants, as well as the numbers of those with neoplasms by 5-year age groups and calendar years for men and women, respectively.

In our study, our calibration targets are the aggregate prevalence values of NON, ADV and CRC of both men and women over five age groups (55-59, 60-64, 65-69, 70-74 and 75-79). Take aggregate prevalence value of NON of men as an example, we can first get five prevalence values for the five age groups which are 16.0% (55-59), 17.2% (60-64), 18.6% (65-69), 19.0% (70-74), 18.2% (75-79). Then the aggregate prevalence value of male NON is $\left( \frac{16.0+17.2+18.6+19.0+18.2}{5} \right)\%=17.80\%$.

**Reference**

1. Brenner H, Altenhofen L, Stock C, Hoffmeister M. Natural history of colorectal adenomas: birth cohort analysis among 3.6 million participants of screening colonoscopy. Cancer Epidemiol Biomarker Prev. 2013;22(6):1043-51.
